# Novel Technology for Unbalance Diagnosis for Dual-Speed Wind Turbines

**DOI:** 10.3390/s26072268

**Published:** 2026-04-07

**Authors:** Amir R. Askari, Len Gelman, Russell King, Daryl Hickey, Mehdi Behzad

**Affiliations:** 1 Department of Engineering, School of Computing and Engineering, The University of Huddersfield, Huddersfield HD1 3DH, UK; l.gelman@hud.ac.uk; 2 Department of Mechanical Engineering, Hakim Sabzevari University, Sabzevar 96179-76487, Iran; 3 Sensonics Ltd., 3 Northbridge Rd., Berkhamsted HP4 1EF, UK; 4 Natural Power, 120 Bath St., Glasgow G2 2EN, UK; darylh@naturalpower.com; 5 Department of Mechanical Engineering, Sharif University of Technology, Tehran 11155-11365, Iran

**Keywords:** signal processing, fault diagnosis, rotating machinery

## Abstract

Unbalance diagnosis for non-constant speed systems is challenging because the 1X fundamental rotational harmonic magnitude, commonly used as an unbalance indicator, depends on shaft rotational speed. This dependency makes it difficult to separate speed effects from unbalance effects. It has been shown that 1X magnitudes become speed-invariant if they are normalized with respect to the rotational speed in power four for variable-speed wind turbines. However, the applicability of this diagnostic technology to dual-speed machines remains unclear. This study experimentally investigates unbalance diagnosis technologies for dual-speed wind turbines, for which speed-dependent interference is present. Vibration data are collected from the main bearings of two dual-speed wind turbines. Novel residual-based, speed-invariant unbalance diagnostic technology is proposed. The experimental results show consistent statistical distributions of the new diagnosis indicator across low and high-speed operating regimes. These findings confirm the suitability of the proposed technology for unbalance diagnosis for dual-speed rotating machinery.

## 1. Introduction

Wind power plants are one of the most important sectors that are responsible for supplying renewable electricity. Wind turbines are operating in harsh environments. Therefore, their rotors are vulnerable to the unbalance phenomenon [1] usually caused by erosion [2,3] or icing [4,5] during operations. Therefore, they need to be continuously monitored against unbalance to prevent additional maintenance costs [6].

The unbalance phenomenon, which is practically unavoidable in rotating machinery, will not be considered as a fault if its severity does not exceed a permissible level [7]. However, due to the large size of wind turbine rotors, the unbalance severity can simply increase and may even cause a catastrophic failure [8]. Besides increasing the level of stress in the turbine components, the unbalance fault affects the machine drive train and causes low-quality power generation as well [9]. Therefore, it is very important to continuously monitor the balance conditions of wind turbines in order to detect any unbalance, higher than the permissible level [7], at an early stage.

Wind turbines are classified into machines operating at (i) fixed-, (ii) dual-, and (iii) variable-speed conditions:

- *Fixed-speed turbines:* These machines start to operate when the wind speed exceeds a certain level [10]. In this situation, regardless of the wind speed variations, the rotor speed is kept constant by a gearbox [10]. These systems benefit from simplicity, robustness, and low maintenance costs [10]. However, they suffer from poor energy conversion efficiency [11]. In addition, sudden changes in the wind speed cause additional mechanical stresses in the gearbox components [12].
- *Dual-speed turbines:* Compared to fixed-speed wind turbines, these machines have the ability to operate at a wider range of wind speeds [13]. This type of wind turbine benefits from low maintenance costs and high energy conversion efficiency simultaneously. That is, they are able to harvest energy above 95% of that scavenged by variable-speed machines, as the most efficient systems available in the wind industrial sector, and at the same time, benefit from the simplicity and robustness of the fixed-speed architectures [14,15,16].
- *Variable-speed turbines:* These machines benefit from adjustable rotational speed according to the wind velocity that causes the highest energy conversion efficiency and low stress on the gearbox components [17]. However, given the requirement for more complex subsystems, they suffer from high maintenance costs [14].

The unbalance fault is caused by the centrifugal force generated by the mass unbalance that rotates with the machine’s rotational speed [18,19,20]. Consequently, it can be diagnosed via monitoring the magnitude of the vibration of the first rotational harmonic [21,22,23,24,25,26]. Since the centrifugal excitation increases proportionally with rotational speed [18], the magnitude of the 1X component of the vibration signal becomes dependent on the rotational speed, which is a key characteristic of this fault [27].

Ramlau and Niebsch [28] assessed the dynamics of unbalanced wind turbines constrained to a fixed rotational speed via the finite element method. Investigating the inverse problem linking the fault severity and the resulting nacelle vibrations, they indicated that the fault is reflected in the 1X magnitudes of the vibration signal. Given the speed dependency of the 1X magnitudes, unbalance diagnosis in systems, operating under fixed-speed conditions is not challenging. However, the diagnosis of systems not operating under constant speed faces a big challenge because the threshold between healthy and faulty conditions is not constant and varies with speed variation.

Li et al. [9] analyzed unbalance-related anomalies in wind turbines operating under variable-speed conditions. They used G.H.Bladed software, version 4.2, to develop a mathematical turbine model and obtain a numerical torque signal. Utilizing the order tracking technique [29,30,31,32,33,34,35], Li et al. [9] identified the ratio between the magnitudes of the first- and third-rotational harmonics as a diagnostic indicator for rotor unbalance. According to the findings presented in the study, the intensity of the fundamental harmonic demonstrates significantly greater sensitivity to changes in rotational speed compared to the third-order harmonic. Consequently, a degree of speed dependence in the proposed diagnostic metric is to be expected. However, this issue is not addressed in this study.

Wu et al. [36] assessed a variable-speed unbalanced disk–shaft system. They adopted the tacholess order tracking method to obtain 1X magnitudes of the vibration signal. They showed that their processing tool is much more accurate in comparison to the short-time Fourier transform. Nonetheless, this aspect is not explored within the scope of this investigation.

Xu et al. [37] examined rotor unbalance in a 750 kW variable-speed wind turbine. To extract the magnitudes of the first three rotational harmonics from the vibration data acquired from the turbine’s drivetrain, they applied the complex Morlet wavelet transform. They assessed the wind turbine health condition via monitoring the ratio between the summation of the magnitudes of the first two rotational harmonics to that of the third one as the fault indicator. The sensitivity of the diagnostic indicator to the unbalance severity is addressed in this study. However, its speed dependency is not assessed.

To mitigate the speed dependency of 1X vibration magnitudes, Askari [38] proposed a speed-invariant diagnostic technology for unbalance detection for variable-speed wind turbines. They analytically derived the relationship between the 1X harmonic magnitude and rotational speed and, based on this dependency, normalized the fundamental rotational harmonic magnitudes with respect to the rotational speed in power four to obtain a speed-invariant unbalance indicator. The effectiveness of the proposed technology is experimentally validated using vibration data collected from a 2.3 MW variable-speed wind turbine. It has been shown that the speed-invariant indicator is also independent of torsional load, making it suitable for unbalance diagnosis for variable-speed wind turbines, irrespective of rotor speed or torsional load [39].

Askari [38,39] estimated the average interference level in the vicinity of the fundamental rotational harmonics and subtracted it from the corresponding 1X magnitudes to obtain residuals. These residuals are subsequently normalized with respect to the average surrounding interference. However, according to the investigation made in Section 4, it is found that since the speed dependency of the local interferences is not negligible, this unbalance technology does not perform adequately for dual-speed wind turbines. Accordingly, the primary objective of the present study is to investigate this limitation and address unbalance diagnosis for dual-speed wind turbines.

Unbalance may occur in different rotating components of a wind turbine, including the low-speed shaft, which is connected to the blades, drivetrain stages, and a generator. Unbalance in these components can also be diagnosed invariantly from shaft speed using the proposed technology. Vibration signals need to be acquired at appropriate locations close to the component of interest, along with the corresponding shaft rotational speed. The indicator of the proposed diagnostic technology could be obtained similarly by dividing the fundamental rotational residual magnitude by shaft speed in power four. It is notable that the present study has specifically been customized for the main wind turbine low-speed rotor because the most vulnerable part of a wind turbine to unbalance fault is its rotor, including the blades.

To the best of the authors’ knowledge, unbalance fault diagnosis for dual-speed wind turbines has not been reported in the existing literature [13,14,15,16]. Therefore, the novelties of the present paper are as follows:

- This is the first worldwide investigation of unbalance fault diagnosis for dual-speed wind turbines and includes:
- Speed-invariant unbalance diagnostic technology for dual-speed wind turbines.
- Experimental trial results of the proposed technology.

In view of the novelties of the present work, the objectives of this paper are as follows:

- To theoretically investigate dependence of 1X harmonic magnitudes on rotational speed in machines operating at multiple constant speeds.
- To collect vibration data at the main bearing of two dual-speed wind turbines operating within a permissible level of unbalance.
- To adopt higher-order STCFT to process vibration data.
- To calculate the average local interference level and to obtain 1X residual and normalized magnitudes.
- To obtain residual and normalized 1X magnitudes as well as their corresponding speed-invariant unbalance indicators.
- To quantify separations between the probability density functions (*PDFs*) associated with the low- and high-speed diagnostic feature histograms by adopting the Fisher criterion (*FC*) and separation probability (*SP*).

The remainder of the paper is organized as follows. Section 2 theoretically obtains speed-invariant diagnosis technology for low-speed machinery operating at multiple constant speeds. Section 3 illustrates experimental setup. The details of diagnostic technology implementation that includes application of higher-order STCFT to process vibration data, calculating the average local interference level, and determining 1X residual and normalized magnitudes, as well as their corresponding speed-invariant indicators, are also presented in this section. The findings are presented in Section 4, while the key conclusions of the study are summarized in Section 5.

## 2. Theoretical Analysis

A rotating body is considered to be dynamically balanced when its central principal axis aligns with its axis of rotation [7,19]. In such a condition, no net centrifugal force acts on body. However, in practical applications, all rotating machinery exhibits some degree of unbalance, making a perfectly balanced state unattainable. Nevertheless, rotating machinery must be balanced according to its specific application and operating rotational speed in order to satisfy the minimum balance quality requirements [7]. Any resultant unbalance exceeding the specified limits for the corresponding balance grade is classified as a rotor unbalance fault.

Dynamics of wind turbine is modeled by the equivalent mass, spring, and damper system. Therefore, in general, dynamics is governed by the three equations corresponding to the three spatial dimensions of $\hat{x}$, $\hat{y}$, and $\hat{z}$, where $\hat{x}$ is aligned with the wind turbine main shaft, pointing outward; $\hat{y}$ is the second horizontal axis, perpendicular to the main wind turbine shaft and pointing to the right; and $\hat{z}$ is the vertical axis that is pointing upward. However, based on the detailed investigation, reported in ref. [38], it is observed that only the $\hat{y}$-axis exhibits a clear unbalance signature, supported by strong speed dependency. Because the centrifugal force coming from blade unbalance only manifests in the $\hat{y}$- and $\hat{z}$-axes: i.e., the axes that are located in the plane of blade rotation. Theoretically, due to the symmetry of shaft cross-section [38], there is no difference between the vertical and horizontal directions within the plane of blade rotation, as both capture the centrifugal force response. However, in practice, axis with lower stiffness exhibits a clearer unbalance signature [38]. Therefore, for clarity and conciseness, equation of motion is presented only along the $\hat{y}$ direction.

In view of the fact, that dual-speed wind turbines mostly operate at one of their constant speeds, assuming stationary operational conditions, the wind turbine rotor transversal motion is governed by [38]

(1) $$\frac{d^{2}\hat{y}}{d{\hat{t}}^{2}}+2\xi \omega_{n}\frac{d\hat{y}}{d\hat{t}}+\omega_{n}^{2}\hat{y}=\frac{me{\hat{\Omega }}_{0}^{2}}{M}cos\left({\hat{\Omega }}_{0}\hat{t}\right)\;,$$

where $\hat{y}$ denotes the transversal displacement along the horizontal axis perpendicular to the wind turbine main shaft in m, $\hat{t}$ is the dimensional time in s, $\omega_{n}$ is the wind turbine rotor natural frequency in Rad/s, *M* and ξ are, respectively, its equivalent mass, in kg, and damping ratio at the data-capturing location, m is the unbalance mass in kg, e is the eccentricity in m, and ${\hat{\Omega }}_{0}$ is the constant rotational speed in Rad/s.

For convenience, the following dimensionless variables are introduced [38]

(2) $$y=\frac{\hat{y}}{e},\;\;t=\omega_{n}\hat{t},\;\;\Omega_{0}=\frac{{\hat{\Omega }}_{0}}{\omega_{n}},\;$$

Adopting the dimensionless variables, given in Equation (2), the normalized governing equation of motion takes form of [38]

(3) $$\ddot{y}+2\xi \dot{y}+y=\lambda \Omega_{0}^{2}cos\left(\Omega_{0}t\right)\;,$$

where the dot sign represents differentiation with respect to the dimensionless time, and the normalized unbalance parameter is given by $\lambda =\frac{m}{M}$.

By considering only a particular solution of Equation (3) and disregarding the homogeneous component, typically attenuated rapidly due to damping [40], the equation yields the following solution [41]

(4) $$y\left(t\right)=\frac{\lambda \Omega_{0}^{2}}{\sqrt{{\left(1-\Omega_{0}^{2}\right)}^{2}+{\left(2\xi \Omega_{0}\right)}^{2}}}cos\left[\Omega_{0}t-{tan}^{-1}\left(\frac{2\xi \Omega_{0}}{1-\Omega_{0}^{2}}\right)\right]\;.$$

As demonstrated in Equation (4), an unbalance fault manifests at the fundamental shaft rotational frequency, i.e., $\Omega_{0}$. It can be diagnosed by monitoring the magnitude of the fundamental rotational harmonic.

In view of the fact, that accelerometers measure the acceleration signal, two times differentiating Equation (4) with respect to time *t* provides the speed dependency of the acceleration magnitude of the fundamental rotational harmonic as [38]

(5) $$C=\frac{\Omega_{0}^{4}}{\sqrt{{\left(1-\Omega_{0}^{2}\right)}^{2}+{\left(2\xi \Omega_{0}\right)}^{2}}}\;\;.$$

As observed, this dependency is governed by the rotational speed, the resonance frequency, and the equivalent damping ratio. However, for low-speed systems, operating under the condition $\Omega_{0}\ll 1$, this relationship can be simplified to [38]

(6) $$C_{simp}=\Omega_{0}^{4}\;\;.$$

It has been substantiated, that this equation works well, with an acceptable accuracy, for large wind turbines, whose rotational speeds are less than or equal to one-fifth of their resonance frequencies [38]. This statement is supported by the fact, that the lowest resonance frequency of the main shaft, made of steel, is 103.05 Hz, estimated by using an in-house finite element code, developed in MATLAB, version R2024a. In addition, as it will be discussed later, the highest rotational speed of an employed wind turbine is 0.275 Hz.

It is notable, that other faults, such as bearing defects, could be identified using model-based condition monitoring, provided, that appropriate bearing dynamic models are employed.

Bearing faults are classified as localized and distributed fault types. Localized faults are typically diagnosed by monitoring their corresponding characteristic defect frequencies, which differ from the fundamental shaft rotational frequency. In contrast, distributed faults, arising from a bearing eccentricity, a bearing misalignment, or an insufficient bearing lubrication [42], influence not only the magnitude of the fundamental rotational harmonic, but also the magnitudes of its higher harmonics. Therefore, reliance on the magnitude of the fundamental harmonic of the shaft rotational frequency alone is insufficient for accurate diagnosis of such faults. Consequently, localized and distributed bearing faults cannot be diagnosed in the same way as an unbalance and require a different analysis, which is beyond the scope of the present study.

## 3. Experimental Setup and Diagnostic Indicator Extraction

### 3.1. Experimental Setup

A dual-speed 2.3 MW wind turbine with a permissible unbalance level below $5.94\times 10^{8}gr\cdot mm$, as specified in [7,8,38,39], is assessed. The turbine is a horizontal-axis configuration with three 40 m blades and is designed to operate at two nominal rotational speeds: 0.185 Hz (low speed) and 0.275 Hz (high speed).

Turbine vibrations, together with the rotational speed of the main shaft and wind speed, are collected via a data acquisition system, installed inside the turbine’s nacelle unit, during a long monitoring period.

**Figure 1:**
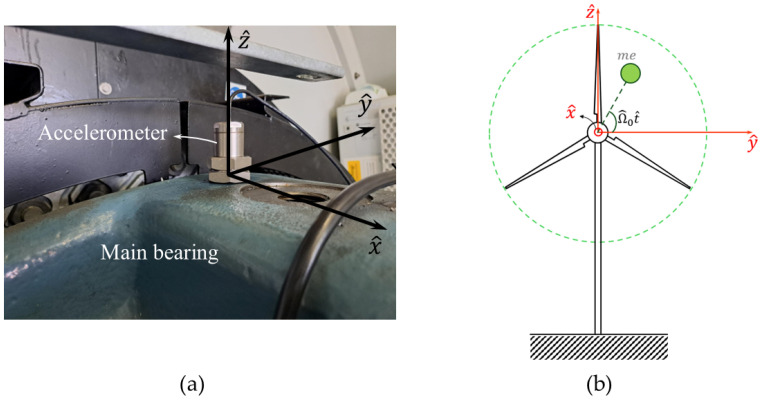
(**a**) Accelerometer installation and (**b**) kinematic representation of accelerometer directions [38].

Figure 1a shows the installation of accelerometer and its orientation. For clarity, Figure 1b provides a kinematic representation of accelerometer directions. As illustrated, accelerometer is mounted on the main bearing of the wind turbine, which is the location closest to the rotor.

**Figure 2:**
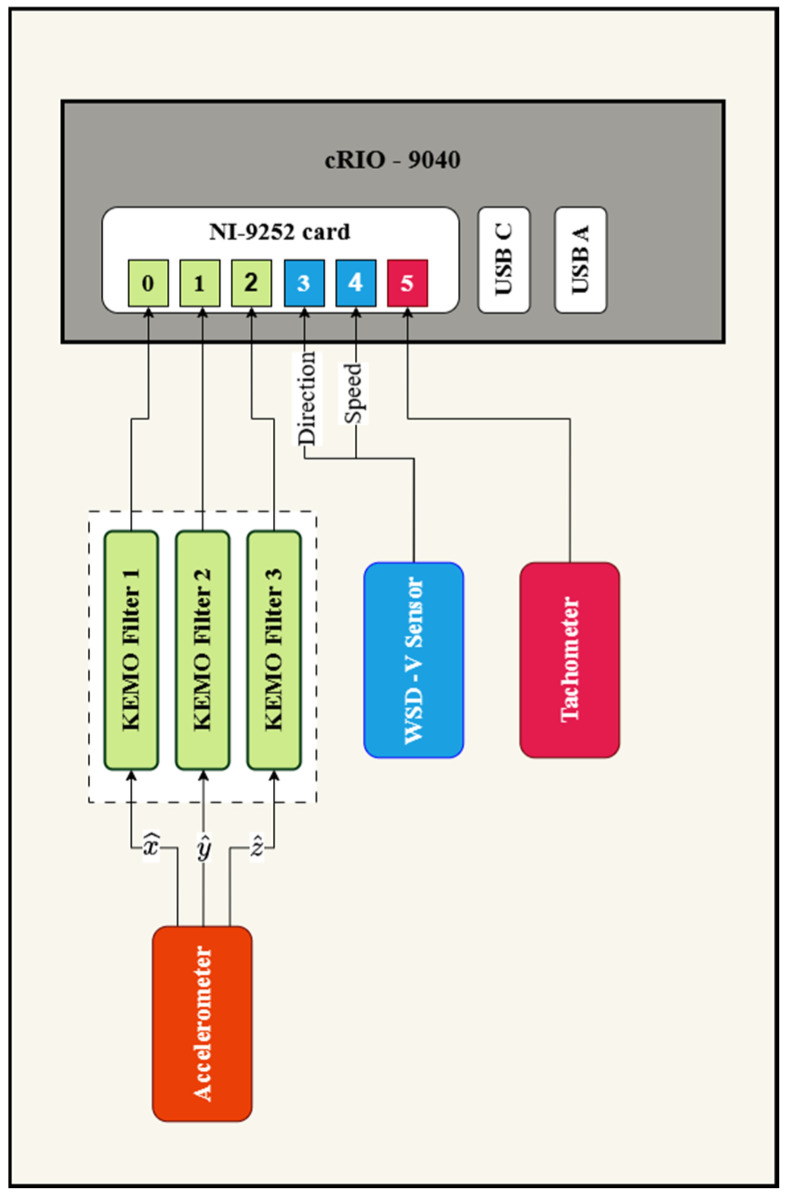
Schematic of the data-capturing system.

Figure 2 illustrates connections between data-capturing components, including: (I) a three-axes MEMS accelerometer (II) three DR 1600 KEMO anti-aliasing filters, (III) an inductive proximity sensor (IV) a wind speed and direction sensor, and (V) a data acquisition unit. Specifications of these components are detailed in Table 1. The detailed wiring diagram of data acquisition system is provided in [38].

### 3.2. Diagnostic Indicator Extraction

In view of the frequent switching of dual-speed wind turbines between low- and high-rotational-speed conditions, and speed fluctuations, that are unavoidable during a turbine operation, the vibration data are processed by the higher-order STCFT. This nonstationary processing tool is proposed by L. Gelman et al. [43] and benefits accurate extraction of harmonic magnitudes in nonstationary conditions [44,45]. This transform states [43] as:

(7) $$V\left(f,\hat{T}\right)=\frac{1}{T}\int_{-\infty }^{+\infty }h\left(t-\hat{T}\right)v\left(t\right)e^{-2\pi j\left(\int_{0}^{t}f\left(\tau \right)d\tau \right)}dt\;,$$

where $j=\sqrt{-1}$, $v\left(t\right)$ denotes signal, f is the instantaneous frequency in Hz and $h\left(t\right)$ is time window with centre of $\hat{T}$ and the duration of *T*.

Adopting the STCFT, the 1X magnitude is obtained. However, to remove the influence of the local interference around the harmonic peak, 1X residual and normalized [38,39] magnitudes, corresponding to an unbalance fault, are obtained. To this end, the average local interference level first needs to be obtained. This is given by [38,39,46,47]

(8) $$N_{ave}=\frac{\sum_{i=1}^{n_{l}}N_{i}^{left}+\sum_{i=1}^{n_{r}}N_{i}^{right}}{n_{l}+n_{r}}\;,$$

where $N_{i}^{left}$ is the intensity of the *i*th frequency component from the peak on its left, $N_{i}^{right}$ denotes a similar parameter for the right side of the peak. $n_{l}$ and $n_{r}$ are the number of points, whose intensities participate in the calculation of the average local interference level.

**Figure 3:**
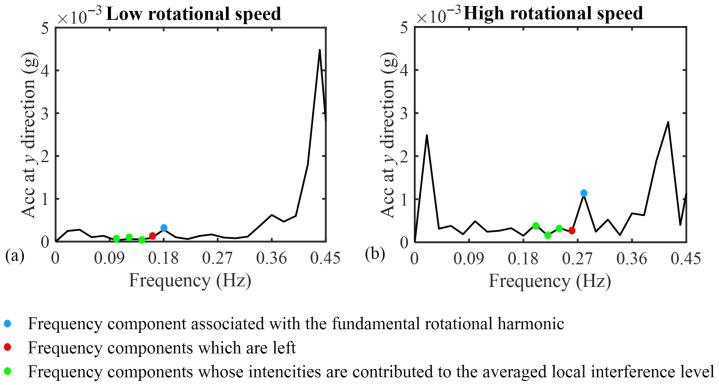
1X magnitude and the magnitudes of local interference in (**a**) low and (**b**) high rotational speed regimes.

Here, as clarified in Figure 3 for both the low- and high-speed regimes, the nearest component to the peak is left, and values of $n_{l}$ and $n_{r}$ are selected as 3 and 0, respectively. The reason behind this selection is that all the components’ intensities after the fundamental peak are affected by the next peak. Therefore, they should not be considered as local interferences.

Having the average surrounding interference level, the residual ($S_{R}$) and normalized ($S_{N}$) peak values, which could be used for unbalance diagnosis, are obtained by [38,39,46,47]:

(9) $$S_{R}=\sqrt{P^{2}-N_{ave}^{2}}\;\;,$$

(10) $$S_{N}=\frac{\sqrt{P^{2}-N_{ave}^{2}}}{N_{ave}}\;\;,$$

where P is the 1X magnitude [48], obtained via the STCFT.

According to the universal theoretical background, outlined earlier, for machines operating at low speeds, the magnitude of the fundamental rotational harmonic in vibration signals changes proportionally to the fourth power of the rotational speed. Therefore, having the 1X residual [49] peak magnitudes, i.e., $S_{R}$, the residual-based speed-independent unbalance indicator, denoted as $S_{NR}$, is proposed here as

(11) $$S_{NR}=\frac{S_{R}}{{\bar{f}}^{4}}\;\;,$$

where $\bar{f}$ corresponds to the average rotational speed across each time window in Hz. Notably, dividing the normalized 1X magnitudes, i.e., $S_{N}$, with respect to the rotational speed in power 4 yields a normalized-based speed-independent unbalance indicator, i.e., $S_{NN}$ [38], as:

(12) $$S_{NN}=\frac{S_{N}}{{\bar{f}}^{4}}\;\;,$$

Notably, $S_{N}$ is dimensionless. In addition, units of $S_{R}$, $S_{NR}$, and $S_{NN}$ are g, g·s^4^, and s^4^, respectively.

## 4. Results and Discussions

Data have been collected from two dual-speed wind turbines across a prolonged timeframe and recorded within 60 min time portions.

Given the stronger unbalance signature on the $\hat{y}$ direction, which is the second horizontal axis perpendicular to the wind turbine main shaft, the vibration data are processed only along that direction [38,39,47]. Data are processed via the STCFT with a 50 s-time window and 40% overlapping. Doing so, the residual and the normalized 1X magnitudes, as well as their corresponding speed-invariant features, are obtained.

The total number of each diagnostic indicator is provided separately for each wind turbine and each operational speed in Table 2. As this table demonstrates, the wind turbines mostly operated at high wind speed conditions, resulting in a higher number of indicators at high-rotational speeds. However, the kinetic energy, associated with low wind speed conditions, which are related to low rotational speeds, is also scavenged. This is the main difference between fixed-speed and dual-speed wind turbines, and causes increase in the energy conversion efficiency of machines [14].

**Figure 4:**
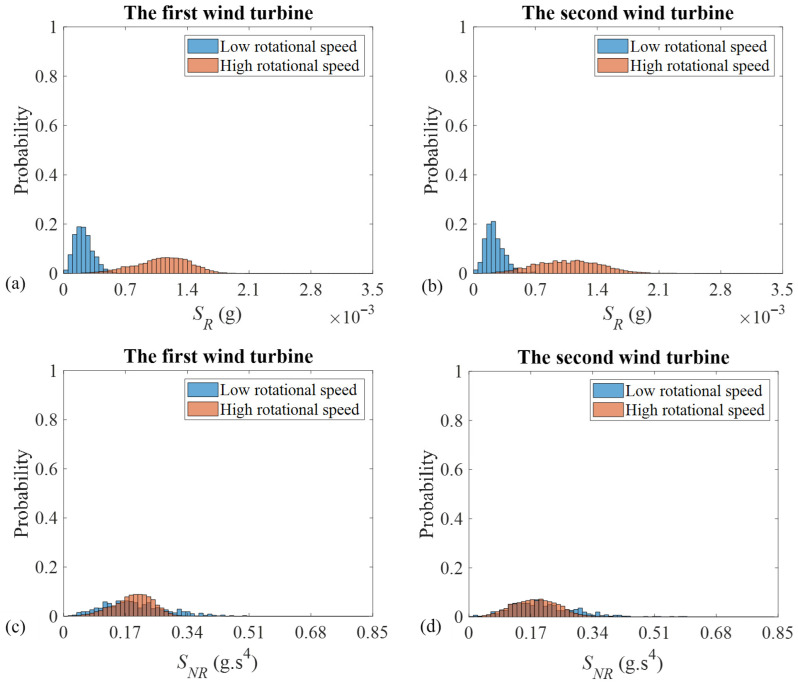
Histograms of the 1X residual magnitudes and their corresponding speed-invariant unbalance diagnostic feature for the first and the second wind turbines: (**a**) the 1X residual magnitudes ($S_{R}$) for the first wind turbine, (**b**) the 1X residual magnitudes ($S_{R}$) for the second wind turbine, (**c**) the proposed feature ($S_{NR}$) for the first wind turbine, and (**d**) the proposed feature ($S_{NR}$) for the second wind turbine.

**Figure 5:**
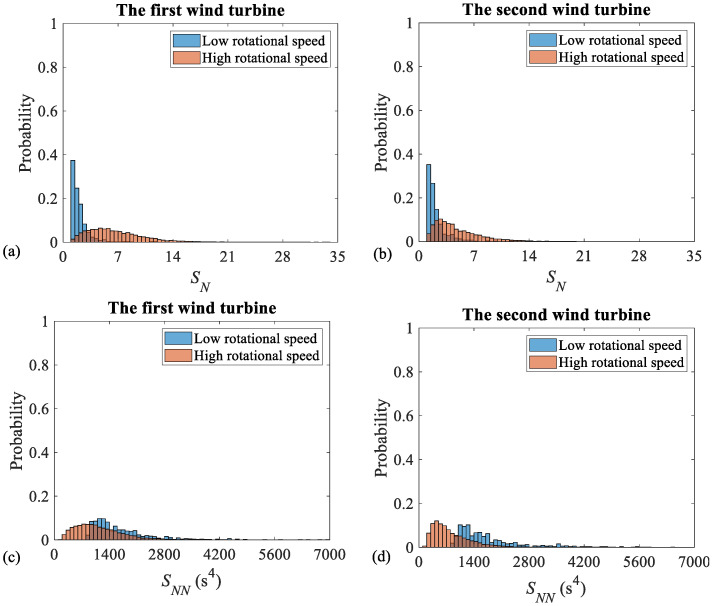
Histograms of 1X normalized magnitudes and their corresponding speed-invariant unbalance diagnostic features for the first and the second wind turbines: (**a**) 1X normalized magnitudes ($S_{N}$) for the first wind turbine, (**b**) 1X normalized magnitudes ($S_{N}$) for the second wind turbine, (**c**) speed-invariant feature based on 1X normalized magnitudes ($S_{NN}$) for the first wind turbine, and (**d**) speed-invariant feature based on 1X normalized magnitudes ($S_{NN}$) for the second wind turbine.

The histograms, associated with the residual and the normalized 1X magnitudes, as well as their corresponding speed-invariant diagnostic indicators, are, respectively, given in Figure 4 and Figure 5 for both the first and the second wind turbines. These figures compare the *PDFs* of low- and high-rotational-speed cases. The histograms representing the low- and the high-rotational speeds are depicted in blue and orange, respectively. The region. in which these two histograms overlap is shown in brown, resulting from the blending of the two colours.

To quantify the separation between the histograms, two measures, the *FC* and the *SP*, are adopted. The *FC* is implemented by [50,51]:

(13) $$FC=\frac{{\left(m_{L}-m_{H}\right)}^{2}}{\mu_{L}^{2}+\mu_{H}^{2}}\;\;,$$

where *m* represents the mean value, and *μ* denotes the standard deviation of diagnostic feature, and the subscripts L and H correspond to the low- and the high-rotational-speed conditions, respectively.

The *SP* is obtained by fitting the normal *PDF* to the histograms of diagnostic feature *F* via [52]

(14) $$PDF\left(F\right)=\frac{1}{\mu \sqrt{2\pi }}\;e^{\frac{-{\left(F-m\right)}^{2}}{2\mu^{2}}}\;\;.$$

Considering two *PDFs*, whose normal estimations are overlapping, an intersection point is chosen as a threshold. Based on the Bayesian theory [51,53], estimate of the *SP* yields [52]

(15) $$P=\frac{F_{L,\;c}+F_{H,\;c}}{F_{L,\;t}+F_{H,t}}\times 100\%\;,$$

where $F_{L,\;c}$ represents count of features from the low-rotational-speed group with values below the threshold, $F_{L,\;t}$ denotes the total number of features in that low-speed group. $F_{H,\;c}$ refers to the number of features from the high-rotational-speed group exceeding the threshold, and $F_{H,t}$ is the total number of features associated with the high-speed group.

In view of Equation (15), if two histograms are fully separated, estimate of the *SP* will approach 100%. The *SP* for fully overlapping histograms leads to 50%. Thus, greater the *SP*, more distance between histograms of two features [52].

The *FC* associated with the residual and the normalized 1X magnitudes, as well as their corresponding speed-invariant unbalance diagnostic indicator across the low- and the high-speed regimes, are given in Table 3. As is seen, the *FC* takes large values for the 1X residual magnitudes and very small values in the vicinity of zero for the speed-invariant diagnostic feature, if it is obtained based on the 1X residual magnitudes. This shows, that the proposed unbalance technology mitigates the speed dependency of the 1X residual magnitudes and benefits from similar *PDF*s over the low- and the high-rotational speeds.

The strong speed dependence of the 1X residual magnitudes indicates, that the observed vibrations are primarily due to permissible rotor unbalance rather than other potential faults, such as bearing local defects, as noted by Rafaq et al. [27], who identify dependence of the 1X magnitudes on the shaft rotational speed as the main signature of a mass unbalance, distinguishing it from other faults, that may also affect the fundamental rotational harmonic. This observation is consistent with wind turbine inspection reports, which confirm that the machines are healthy, without any bearing defects, and operating with permissible levels of unbalance.

Bearing local defects are typically characterized by the distinct fundamental defect frequencies, namely BPFO (Ball Pass Frequency Outer race), BPFI (Ball Pass Frequency Inner race), BSF (Ball Spin Frequency), and FTF (the Fundamental Train Frequency, also called Cage Frequency), and higher-order defect frequencies [52,54,55]. These frequencies usually are not integer multiples of the shaft rotational speed; therefore, it is highly unlikely that the defect frequencies, generated by bearing local defects, coincide with the fundamental shaft rotational speed. Notably, in a rare case of a very specific bearing, in which the bearing defect frequencies become integer multiples of the shaft rotational frequency, both faults, i.e., a bearing defect and an unbalance, would be reflected in the same fundamental shaft rotational frequency harmonic. However, this scenario is highly unlikely and does not correspond to the present study.

As Figure 5 demonstrates, despite the residual-based technology, the one on the basis of the normalized indicators is not effective. As it is seen, the normalized 1X magnitude, i.e., $S_{N}$, does not exhibit the expected strong speed dependency. Also, the speed-invariant indicator based on 1X normalized magnitudes [38], i.e., $S_{NN}$, shows inconsistent *PDF*s across the low- and the high-speed regimes: the *FC* for the first and the second wind turbines take the non-negligible values of 0.22 and 0.68, respectively. It is noteworthy that, for variable-speed wind turbines, this parameter is reported to take values that are effectively equal to zero, up to three decimal places [38].

These results indicate that, for dual-speed wind turbines, the unbalance technology, based on the normalized 1X magnitudes, is ineffective. Consequently, the residual-based technology emerges as the only reliable approach for an unbalance diagnosis in this class of wind turbines.

Table 4 provides the estimate of the *SP* for the 1X residual and normalized magnitudes, as well as their corresponding speed-invariant unbalance diagnostic features. As this Table demonstrates, the *SP* takes values close to 100% for the 1X residual magnitudes and leads to 50% for the case of the proposed speed-invariant diagnostic feature. This observation, which agrees excellently with those, obtained based on the *FC* assessment in Table 3, indicates, that the proposed unbalance diagnostic technology, based on the adoption of the 1X residual magnitudes, is speed independent. Therefore, this technology can successfully be applied for an unbalance diagnosis for dual-speed wind turbines.

In contrast, the 1X normalized magnitude, i.e., $S_{N}$, exhibits weak speed dependence. In addition, its corresponding speed-invariant feature, i.e., $S_{NN}$, faces a limited speed independence. These findings, which are consistent with the conclusions, drawn from the *FC* analysis in Table 3, further demonstrate, that only the proposed residual-based unbalance technology successfully mitigates the inherent speed dependency of the 1X magnitudes and provides a stable statistical distribution across both the low- and the high-rotational-speed regimes.

**Figure 6:**
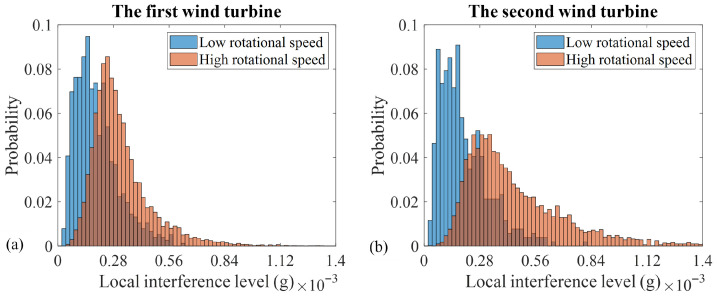
Histograms of the local interference level for (**a**) the first and (**b**) the second wind turbines.

To elucidate why the normalized-based technology, shown to perform effectively for variable-speed wind turbines [38], is not suitable for dual-speed machines, Figure 6 presents histograms of the average local interference level for the low- and the high-rotational-speed regimes of both wind turbines. The corresponding *FC* values and estimates of the *SP* between the low- and the high-speed *PDF*s, shown in Figure 6, are summarized in Table 5.

As indicated by Figure 6 and Table 5, the local interference level exhibits a non-negligible dependence on rotational speed. Consequently, normalizing the 1X residual magnitudes with respect to the local interference level alters their inherent speed dependence. As a result, further normalization by the rotational speed in power four, intended to obtain the speed-invariant unbalance indicators [38], becomes ineffective, yielding technology, that is no longer speed-invariant. Therefore, in scenarios, in which the local interference level is speed-dependent, like in dual-speed wind turbines, use of the residual-based technology is required to ensure robust and speed-independent unbalance diagnosis.

Considering the findings discussed above, use of the proposed speed-independent diagnosis technology, based on the 1X residual magnitude, is strongly recommended for diagnosing an unbalance for dual-speed wind turbines. Its indicator maintains consistent values across both the low- and the high-rotational-speed conditions and exhibits a sensitivity solely to severity of an unbalance fault. This means, that regardless of the level of the rotational speed, one can monitor this fault indicator to judge a balance condition of dual-speed wind turbines.

## 5. Conclusions

This paper presents the first worldwide investigation on an unbalance fault diagnosis for dual-speed wind turbines. In view of the inability of the normalized diagnostic technology, introduced in [38], for dual-speed wind turbines, the novel unbalance speed-invariant diagnostic technology, based on the residual magnitude, is proposed for dual-speed wind turbines.

Diagnostic effectiveness of both the normalized and the proposed speed-invariant diagnostic technologies is evaluated using the vibration data, collected from the two dual-speed wind turbines. The vibration data are processed by the higher-order STCFT, and efficiency of the diagnostic technologies is examined by the Fisher criterion as well as by the separation probability between the low- and the high-rotational-speed indicator histograms.

It is found that the normalized diagnostic technology is not speed-invariant for the dual-speed wind turbines, in contrast to what has been reported for variable-speed wind turbines [38].

In contrast to the normalized-based technology, it is obtained, that the proposed technology is speed-invariant for the dual-speed wind turbines. The Fisher criterion for the proposed speed-invariant unbalance diagnostic feature takes values of 0.0005 and 0.02 for the first and the second wind turbines, respectively. Their corresponding estimates of the separation probability are also very low and close to 50%.

These results demonstrate that, despite the normalized technology, the proposed one performs effectively for the dual-speed wind turbines, producing the consistent probability density functions across the low and the high-speed operating regimes.

This observation is attributed to the non-negligible speed dependency of the local interference level in the dual-speed machines, whereby the normalization by the local interference alters the inherent speed dependency of the fundamental rotational harmonics and inhibits the achievement of true speed invariance after division by the rotational speed in power four.

The findings highlight, that the proposed diagnostic technology is effective for an unbalance diagnosis for important industrial cases, for which the local vibration interference is speed-dependent, as in dual-speed wind turbines. This finding suggests, that the proposed technology could be effectively applied to other rotating machinery with speed-dependent local vibration interference across different industrial sectors.

The results of this study are considerable for an unbalance diagnosis for rotating machinery with speed-dependent local vibration interference. The proposed residual-based technology presents a novel conceptualization and will make a considerable impact on an unbalance diagnosis in mechanical engineering, electrical engineering, vibration diagnosis, etc.

## Figures and Tables

**Table 1:** Data acquisition system components and specifications.

Components		Model/Type	Key Specifications	Operational Conditions	Notes
Accelerometer		PCB 3743F112G—sourced from PCB Piezotronics Inc., Hertfordshire, UK	Triaxial MEMS accelerometer Sensitivity: 1350 mV/g Measurement range: ±4 g Frequency range: 0–1.5 kHz Nonlinearity: 0.1% Transverse sensitivity: ~1%	Temperature range: −54 °C to +121 °C Temperature-dependent sensitivity variation: ±1%/°C	Axes orientation: Same as shown on Figure 1b Powered via USB-A port on the DAQ chassis
Anti-aliasing filters		DR 1600 KEMO (×3)—sourced from Kemo Limited, Thetford, UK	Adjustable analog filters Gain: 10 Cut-off frequency: 10 kHz Power input: 3 W Total harmonic distortion: <0.003%	Temperature range: −10 °C to 45 °C	Used to amplify and band-limit accelerometer signals before digitization
Rotational speed sensor		LJ12A3-4-Z/BY inductive proximity sensor—sourced from Yuanhuang Electric Technology Co., Ltd., Wenzhou, China	32 pulses per revolution Response frequency: 500 Hz Detection range: 4 mm	Temperature range: −25 °C to 55 °C	Measures the main shaft rotational speed
Wind speed and direction sensor		MSL WSD-V—sourced from Measurement Systems Ltd., Newbury, UK	Wind speed range: 0.5–50 m/s Resolution: <0.5 m/s	Temperature range: −20 °C to 60 °C	Wind speed/direction recorded for other purposes, not used in the present analysis
Data acquisition unit	DAQ module	NI-9252 (Web DAQ)—sourced from National Instruments Corporation (UK) Ltd., Newbury, UK	Eight analog input channels Resolution: 24-bit Max sampling rate: 50 kS/s Used sampling rate: 25 kS/s	Temperature range: −20 °C to 52 °C	Installed in the cRIO-9040 chassis
	DAQ chassis	cRIO-9040—sourced from National Instruments Corporation (UK) Ltd., Newbury, UK	Supports I/O frequencies up to 20 MHz	-	-
	Data storage	External hard drive	-	-	Connected via USB-C port on cRIO-9040

**Table 2:** Number of unbalance diagnostic indicators.

	The First Wind Turbine	The Second Wind Turbine
Low speed (0.185 Hz)	804	555
High speed (0.275 Hz)	8760	5260
Total	9564	5815

**Table 3:** The *FC* between the low- and the high-rotational speeds for the unbalance diagnostic indicators.

		The First Wind Turbine	The Second Wind Turbine
The residual indicator	1X magnitudes	7.34	4.66
	Speed-independent	0.0005	0.02
The normalized indicator	1X magnitudes	1.22	0.65
	Speed-independent	0.22	0.68

**Table 4:** Estimates of the *SP* between the low- and the high-rotational speeds for the unbalance indicators.

		The First Wind Turbine	The Second Wind Turbine
The residual indicator	1X magnitudes	97.91%	96.28%
	Speed-independent	53.97%	54.91%
The normalized indicator	1X magnitudes	84.27%	71.41%
	Speed-independent	59.06%	69.76%

**Table 5:** The *FC* and the estimate of the *SP* between the low- and the high-rotational-speed local interference levels.

	The First Wind Turbine	The Second Wind Turbine
FC	0.42	0.84
SP	65.33%	72.24%

## Data Availability

Data are unavailable due to privacy restrictions.

## Abbreviations

The following abbreviations are used in this manuscript:

DAQ	Data Acquisition
BPFI	Ball Pass Frequency Inner race
BPFO	Ball Pass Frequency Outer race
BSF	Ball Spin Frequency
FC	Fisher criterion
FTF	Fundamental Train Frequency
PDF	Probability density function
SP	Separation probability
STCFT	Short-time chirp Fourier transform
